# Successful Treatment of Hemophagocytic Lymphohistiocytosis Associated with Lupus Nephritis by Using Mycophenolate Mofetil

**DOI:** 10.1155/2017/4159727

**Published:** 2017-09-14

**Authors:** Takashi Nawata, Makoto Kubo, Kosaku Shiragami, Yukinori Nakamura, Masafumi Yano

**Affiliations:** ^1^Department of Medicine and Clinical Science, Division of Cardiology and Clinical Immunology, Yamaguchi University Graduate School of Medicine, Ube, Japan; ^2^Division of Endocrinology, Metabolism, Hematological Science and Therapeutics, Yamaguchi University Graduate School of Medicine, Ube, Japan

## Abstract

An estimated 0.9% to 2.4% of patients with systemic lupus erythematosus (SLE) also have hemophagocytic lymphohistiocytosis (HLH). HLH associated with autoimmune diseases is often refractory to corticosteroid treatment; thus, additional immunosuppressive drugs, such as cyclosporine, cyclophosphamide, or tacrolimus, are required. Here, we describe the case of a 44-year-old Japanese woman who developed HLH associated with lupus nephritis. Initially, her HLH was refractory to treatment with a corticosteroid, tacrolimus, and mizoribine. However, alternative treatment with a corticosteroid, mycophenolate mofetil, and tacrolimus improved both her HLH and lupus nephritis. This case suggests the possibility of mycophenolate mofetil as a key drug for treating HLH associated with SLE.

## 1. Introduction

Hemophagocytic lymphohistiocytosis (HLH) is a disorder characterized by activation of macrophages and/or histiocytes with prominent hemophagocytosis in bone marrow and various organs, such as the lymph nodes, liver, and spleen [1]. Clinical features of HLH include a high fever, cytopenia, hepatosplenomegaly, elevated levels of liver enzymes, and high serum levels of ferritin and triglyceride [2]. HLH is divided into primary and secondary types. Secondary HLH may be caused by several conditions, including viral infections, hematologic malignancies, and autoimmune diseases [2]. Regarding HLH associated with autoimmune diseases, systemic lupus erythematosus (SLE) is the most commonly associated disease [1]. In fact, an estimated 0.9% to 2.4% of patients with SLE also have HLH [1]. Although corticosteroids are the most common treatment for HLH associated with autoimmune diseases, more than half of the patients show resistance to such therapy. Other immunosuppressive drugs, such as cyclosporine, cyclophosphamide, or tacrolimus, are second-line treatments for HLH associated with autoimmune diseases [1, 2]. However, there are few reports regarding the treatment of HLH associated with SLE using mycophenolate mofetil (MMF) [3–5]. Furthermore, there has been one case report about the combined therapy with MMF and tacrolimus to HLH with SLE [5]. Here, we describe the case of a 44-year-old Japanese woman who had HLH associated with lupus nephritis. Although her HLH was refractory to treatment with corticosteroids, tacrolimus, and mizoribine, alternative treatment with a corticosteroid, MMF, and tacrolimus improved both her HLH and lupus nephritis.

## 2. Case Presentation

A 44-year-old Japanese woman was admitted to our hospital because of a 9-month history of abnormal urinalysis results and a 1-month history of cytopenia. She was diagnosed as having SLE at another hospital when she was 20 years old. Six years before this admission, she was once admitted to our hospital and diagnosed as having reactive SLE due to a high-grade fever, hypocomplementemia, thrombopenia, and pericarditis. At this time, she was also diagnosed as having Sjögren syndrome, based on positive results for anti-SS-A antibodies and a positive gum test result. She also had a history of a previous adverse drug reaction to cyclosporine. Before this admission, she was taking a regimen of methylprednisolone (22 mg/day), tacrolimus (trough levels 5–10 ng/mL), and mizoribine (150 mg/day).

On admission, she did not have a fever, and her other vital signs were normal. Although the physical examination revealed joint swelling in the fingers and cutaneous lesions, including butterfly erythema and a subcutaneous hematoma in both lower extremities, no other abnormal findings were noted. The neurologic examination also did not show abnormal findings. Laboratory examinations demonstrated cytopenia (hemoglobin level, 12.3 g/dL; leukocyte count, 30.8 × 10^8^/L; platelet count, 9.2 × 10^10^/L) and high serum levels of ferritin, soluble interleukin-2 receptor, and D-dimer (3641 ng/mL, 3864 U/mL, and 12.1 mg/L, resp.). Her levels of hepatobiliary enzymes were elevated (aspartate aminotransferase level, 113 U/L; alanine aminotransferase level, 121 U/L; lactate dehydrogenase level, 813 U/L; alkaline phosphatase level, 423 U/L). A high serum triglyceride level and a low serum fibrinogen level were also noted (510 mg/dL and 102 mg/dL, resp.). Her serum C-reactive protein level and erythrocyte sedimentation rate were within normal limits (0.04 mg/dL and 8 mm at 1 hour, resp.). Antinuclear antibodies were positive, but anti-double-stranded DNA (dsDNA) antibodies and anti-Sm antibodies were negative. Low complement levels were not observed (C3 level, 112.9 mg/dL; C4 level, 59.0 mg/dL). Her serum creatinine level (0.90 mg/dL) suggested mildly impaired renal function, and urine sediment analysis revealed abnormal urinary casts, including red blood cell casts, fatty casts, pigment casts, and waxy casts. Her protein-to-creatinine ratio in a spot urine sample was 2.87, suggesting severe proteinuria. The computed tomography scan showed mild hepatosplenomegaly.

**Figure 1:**
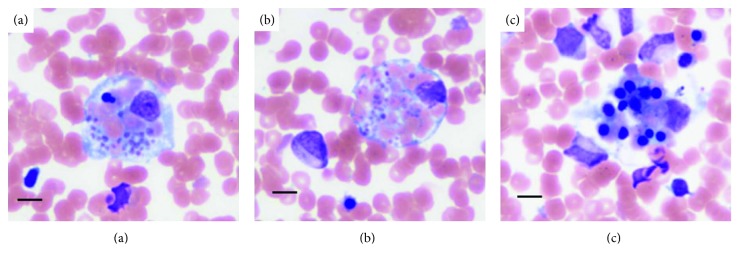
(a–c) Bone marrow examination showing normocellularity and hemophagocytosis without atypical cells (hematoxylin and eosin staining). Scale bars: 20 µm.

Although the oral administration of aspirin as antiplatelet therapy for hip replacement operation prevented our ability to perform a renal biopsy, we diagnosed the patient as having lupus nephritis based on the Systemic Lupus International Collaborating Clinics criteria for SLE [6]. A subsequent bone marrow examination showed normocellularity and hemophagocytosis without atypical cells (Figures 1(a)–1(c)). Cytomegalovirus antigenemia test results were negative, and serology results were negative for Epstein-Barr virus immunoglobulin (Ig) M, parvovirus B19 IgM, and hepatitis viruses B and C. Thus, according to the 2004 HLH diagnostic criteria [7], she was additionally diagnosed as having HLH associated with lupus nephritis.

After diagnosis, she underwent 3 days of steroid pulse therapy (methylprednisolone, 1 g/day), with the oral dose of methylprednisolone increased to 32 mg/day. However, her urinary findings did not improve. Moreover, her cytopenia worsened and serum ferritin level increased further. We considered that the effectiveness of mizoribine was mild, so we switched from mizoribine to MMF for the intensification of treatment for lupus nephritis. Thus, alternative treatment with oral methylprednisolone, MMF (500 mg/day), and tacrolimus was started. After gradual loading of MMF (up to 1500 mg/day), her serum ferritin level and soluble interleukin-2 receptor level decreased within normal limits, and her cytopenia improved. In addition, her liver enzymes and serum creatinine level decreased within normal limits, and her abnormal urinary casts and proteinuria improved (protein-to-creatinine ratio in a spot urine sample was 0.77).

## 3. Discussion

HLH associated with SLE tends to be refractory to treatment with corticosteroids and often requires additional immunosuppressive therapy [2]. MMF is an immunosuppressive prodrug of mycophenolic acid that reversibly inhibits inosine monophosphate dehydrogenase, leading to decreased B cell and T cell proliferation and decreased antibody production [8]. Recently, MMF, instead of cyclophosphamide, has been used as a first-line drug for treating lupus nephritis [9, 10]. However, there are few reports regarding the treatment of HLH associated with SLE using MMF [3–5]. In our case, MMF improved the patient's lupus nephritis and her HLH. This case suggests the usefulness of MMF for HLH associated with SLE. It has been reported that immune cells, especially CD8^+^ T cells and macrophages, play an important role in the pathogenesis of HLH [11]. However, our case in which MMF was effective suggests the possibility that B cells also are related to the pathogenesis of HLH associated with SLE. Recently, it has been hypothesized that B cell–T cell interaction may play an important role in the pathogenesis of SLE [12]. Additionally, autoantibody-mediated and immune complex-mediated mechanisms, both of which are related to B cells, have also been suggested as possible mechanisms for autoimmune-associated HLH [13]. These mechanisms were reasonable in the previous case series reported by Paliga et al., in which the patients showed a high serum level of anti-dsDNA antibodies and low complement levels [3]. However, our patient had normal serum levels of anti-dsDNA antibodies and complements. These findings did not strongly suggest autoantibody-mediated and immune complex-mediated mechanisms. Although activated SLE often showed increasing anti-dsDNA antibodies and low complements, our patient did not have these signs, regardless of activated lupus nephritis [14]. On this standpoint, there is the possibility that pretreatment, especially with methylprednisolone and mizoribine, may work to some extent, and MMF may further affect autoantibody-mediated and immune complex-mediated mechanisms.

Regarding other mechanisms, Sugiyama et al. reported a patient with HLH and SLE who was treated with MMF. In their case report, anti-dsDNA antibodies and complements were within normal limits. They hypothesized that interleukin-18 may have an important role in the pathogenesis of HLH with SLE. Based on their hypothesis, MMF may improve HLH with SLE through inhibiting interleukin-18 and subsequent production of interferon-γ [5]. However, the activity of SLE in their patient was low, which differed from that in our patient who had activated lupus nephritis. As mentioned above, MMF may affect several mechanisms in patients with HLH and SLE. The complicated findings, coexistence of activated lupus nephritis, low level of autoantibodies, and normal level of complements in our patient, which differ from findings reported previously, suggest that this hypothesis may have merit. Although long-term outcomes and the mechanism remain uncertain, MMF may be a key drug for treating HLH associated with SLE. Further accumulation of case reports and studies is required.
